# Deformation Behavior of Single Carbon Fibers Impregnated with Polysulfone by Polymer Solution Method

**DOI:** 10.3390/polym15030570

**Published:** 2023-01-22

**Authors:** Andrey A. Stepashkin, Hussam Mohammad, Elena D. Makarova, Yulia V. Odintsova, Alexander I. Laptev, Victor V. Tcherdyntsev

**Affiliations:** Laboratory of Functional Polymer Materials, National University of Science and Technology “MISIS”, Leninskii prosp, 4, 119049 Moscow, Russia

**Keywords:** single carbon fibers, polysulfone, tensile test, loading rate, structure

## Abstract

Tensile deformation behavior of continuous high-strength and high-modulus single carbon fibers impregnated with a polysulfone solution was investigated. The effect of the carbon fiber type, mass fraction of the polymer, and the loading rate on the tensile strength was studied. It was observed that, whereas for high-modulus carbon fibers the magnitude of tensile strength depends significantly on the loading rate, for high-strength carbon fibers, such dependence was nearly not observed. SEM study shows that at low loading rates, elementary filaments inside the impregnated fiber are able to align themselves along the load application axis because a thermoplastic matrix can flow under the tensile stresses’ force. As a result, the fiber’s strength properties can be realized more effectively in the thermoplastic-based composites than in the same composite with an epoxy matrix.

## 1. Introduction

Carbon fibers with low weight, high strength, high elastic modulus, high thermal conductivity, and low linear expansion were first produced industrially in the 1960s, opening the door for the development of new composite materials and constructions that improve quality of life. Progress in the building, shipbuilding, automobile, and aviation sectors and the use of renewable energy sources has been facilitated by the use of composite materials with polymer matrices reinforced with high-strength fibers [1,2,3,4]. As a result, blades for wind turbines with a length of more than 150 m can now be made using composite materials reinforced with continuous carbon and glass fibers. The maximum power of a single wind generator has thus far reached 12–15 MW for those installed at sea and 7–8 MW for those installed on land [5,6,7].

The use of fibrous polymer composites, while maintaining high reliability, helped to significantly reduce the weight of the airframe, wings, and tail, which made it possible to reduce fuel consumption, reduce CO_2_ emissions, and reduce operating costs [5]. In the designs of passenger aircraft of the latest generation, the share of fibrous polymer composites reaches 50% [8,9].

These days, the most popular composites are polymer composite materials that use thermosetting binders, such as epoxy resins, to create matrices. The fastest advancements in construction, renewable energy, and aviation technology are all related to their utilization. Epoxy binders can achieve high quality impregnation of fiber blanks thanks to their good fluidity and deformation at failure that is equivalent to that of elementary (filament) carbon and glass fibers, which allows for the realization of a major portion of the latter’s strength in structures. Low impact strength and crack resistance, as well as a lengthy technological cycle for product production that can take tens of hours, are drawbacks of thermosetting binders [10,11,12,13].

High-end thermoplastics binders, such as polysulfone (PSU), polyphenylene sulfide, polyethersulfone, and polyetheretherketone, have become more widely available thanks to the development of industrial production, allowing the production of composite materials with high thermal and chemical resistance, high shear strength, resistance to impact loads, and increased crack resistance. In this case, the production cycle can be reduced to a few minutes, creating possibilities for mass manufacture of parts, such as in the automotive sector. In addition, the fact that it is recyclable and reused is another benefit [14,15,16,17,18].

Injection molding, extrusion, and hot forming are the three most common procedures used to create items made of thermoplastic polymers, which differ from those made of thermosetting matrices in terms of technology [19,20,21]. The high viscosity of these polymers’ melts requires the employment of temperatures between 250 and 370 °C and pressures up to 30 bar in order to achieve high-quality impregnation of fiber preforms. This is a characteristic of the used technologies. These processing techniques cause the fibrous preform to face distortion, and the reinforcing fibers may sustain some degree of damage [22,23]. The polymeric material may gradually degrade at processing temperatures above 250 °C as a result of oxidation. The high chemical inertness of thermoplastics reduces the adhesive strength of the “polymer-filler” interface, which decreases the degree of application of the reinforcing fibers’ qualities and lowers the strength characteristics of the resulting composite [24,25]. The features of the deformation behavior of thermoplastic matrices not only predetermine their obvious advantages, but also create a number of serious problems that limit their use, which include a lower degree of implementation of the elastic-strength characteristics of reinforcing fibers compared to epoxy plastics [26,27].

Elementary carbon and glass filaments are characterized by deformations upon destruction of less than 1%, and deformations lie in the range from 1 to 5% in fibers consisting of 1000–24000 filaments, the elongation at destruction due to the sliding of individual filaments in the absence of interaction along the side surfaces. In this case, the deformations of thermoplastic polymers acting as a matrix, at which their flow begins, lie in the range from 1 to 20%, and the flow occurs at stresses at the level of 40–100 MPa [28,29].

A large number of studies have been devoted to the study of the strength characteristics of composite materials based on carbon fibers, since the issues of the formation of strength properties are decisive for understanding the possible applications of composites in engineering. There are a large number of studies devoted to composite materials based on epoxy matrices reinforced with Toray T700 fibers, in which the issues of macroscopic deformations and fracture processes, strength anisotropy, the effect of shear stresses and adhesive interaction on the nature of destruction, and the level of implementation of the strength characteristics of the fiber in the composite are considered [30,31,32,33,34,35,36].

At present, due to the development of research methods, the number of studies of the processes of accumulation and development of damage directly in the process of applying the load in situ is growing, which expands our understanding of the development of deformation processes in composite materials [37,38,39,40].

The use of high-resolution synchrotron computed tomography (SRCT) provides a direct study of fracture processes from the inside in the appropriate micromechanical length scales, in contrast to previously used indirect and/or surface methods. It is shown that fiber breaks are the dominant mechanism of composite damage, and matrix damage, such as transverse cracks and delamination cracks, appears in epoxy matrices before the onset of intense fiber failure. [38]

In [39], using synchrotron X-ray correlation of digital images and computed tomography, T700 carbon fiber/epoxy resin composites were studied under load at different angles to the fiber laying direction. Tensile strength decreases with increasing axial angle. Mapping of the strain field demonstrates the localization of strains at the fiber–matrix interface at large angles of deviation from the axis. It is shown that, depending on the angle of application of the load, the fracture mode changes from predominantly tension (fiber failure) to in-plane shear (separation of the interface).

It was shown using nanoscopic SRCT that for CFRPs with epoxy matrices, the damage distribution in a significant area depends on the local distribution of the matrix thickness between the fibers. It is noted that the destruction occurs in the area where the number of fibers is not large, and the matrix interlayers are large, i.e., in weak areas [40].

At the moment, there is an active study of the deformation behavior of composite material thermoplastic polymer-carbon fiber, interest in which is largely due to the development of additive technologies [8,16,41,42,43,44,45]. In a number of works, composite materials based on thermoplastics and thermoplastics are compared. In [46], the deformation behavior of composites based on polyetheretherketone (PEEK) and epoxy resins reinforced with carbon fiber having the same volume fraction of carbon fiber, about 65%, was studied, including cyclic tests. The PEEK composite showed superior mechanical performance at high cyclic strains compared to epoxy composites due to the microscale shear strain that is realized in PEEK. This is due to the high interfacial strength of the fiber matrix to the final failure in PEEK, in contrast to extensive microcracks, and failure of the fiber–matrix bond in the epoxy composite. The use of a thermoplastic epoxy resin [47] as a CF composite matrix has shown that there is no linear relationship between tensile strength and strain rate. Analysis of the fracture surface shows the presence of ductile fractures, which affect the nature of deformation and fractures of the composite. It concludes that in the case of a thermoplastic matrix, even at relatively low stresses, high adhesive strength at the interfaces might still result in plastic flow of the binder in regionally overstressed areas, which is unsuitable for loaded structural elements [48]. It is important to understand the deformation behavior of composite materials constructed using thermoplastic matrices in order to improve their structure, develop new production techniques, and better understand the outcomes of calculations performed on composite material critical structures.

Previously [49], we applied the melt impregnated technology to form the single carbon fiber composite with a polysulfone matrix. However, as it was shown in [50], solution impregnation technology allows for the significant improvement of the quality of fiber-reinforced composites in relation to the melt impregnation one. In present study, we applied the solution impregnation method to form the single carbon fiber composites, which are suitable test objects to analyze the mechanical behavior of both the couple fiber and individual filaments in the thermoplastic matrix. 

## 2. Materials and Methods

### 2.1. Materials

Samples of high-strength and high-modulus carbon fibers impregnated with the thermoplastic polymer polysulfone were used as the subject of this study. Carbon yarns without a binder and yarns treated with epoxy resin served as comparison samples.

Microcomposites were created using Polysulfone (PSU) Ultrason S 2010 (BASF, Ludwigshafen, Germany) as the matrix material. Due to its high strength properties, excellent impact strength, crack resistance and dimensional stability, and high thermal and chemical resistance, this polymer is used to produce high engineering composites used in the aircraft and automotive industries. Amorphous polymer Polysulfone Ultrason S 2010 has a glass transition temperature of 187 °C, a density of 1.24 g/cm3, a tensile strength at break of 75.0 MPa, and a tension modulus of 2.60 GPa.

Products made from unfilled polysulfone are commonly produced using injection molding, extrusion, or hot forming. When preparing composite materials based on unidirectional carbon tapes and fabrics, it is difficult due to the high melt viscosity of 90 cm3/10 min (360 °C/10 kg). This is because, when impregnated with high-viscosity melts, the fibrous preform undergoes distortions in the laying of the filler, which reduces the strength of the resulting finished product.

The technology elaborated in [50], in which carbon filaments were impregnated with Ultrason S 2010 polysulfone dissolved in n-methylpyrrolidone, followed by removal of the solvent, was used in this work to obtain samples with characteristics comparable to those obtained by impregnation with thermosetting binders, for example, epoxy resins. The applied impregnating solutions of Ultrason S 2010 in N-methylpyrrolidone (CAS: 872-50-4, Molar mass: 99.13 g/mol, Empirical Formula C5H9NO) with concentrations of 20, 30, and 40 wt.% were obtained by mixing the polymer with a solvent in closed quartz flasks at a temperature of 50 °C for 24 h using a magnetic stirrer. The use of polymer solutions with different concentrations made it possible to obtain composite rods with a carbon fiber content of 50 to 90 wt.%.

Toray T700SC–12K (Toray Industries, Inc., Tokyo, Japan), UMT49-12K, and UMT400-12K-EP (Umatex, Moscow, Russia) carbon fibers were used to prepare the composites. Table 1 shows the properties of the used fibers.

### 2.2. Composites Preparation

**Figure 1:**
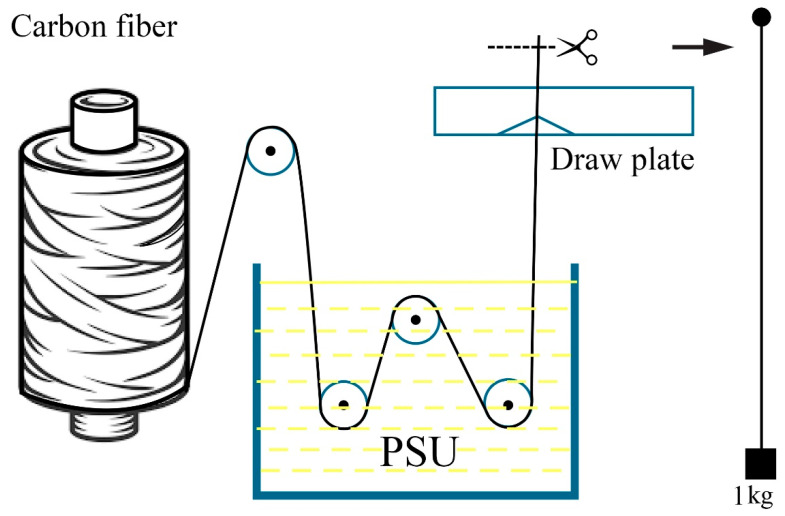
Scheme of impregnation of carbon filaments with a solution of polysulfone and obtaining microcomposites.

Thermoplastic microcomposites were impregnated in a cuvette that contained a polysulfone in n-methylpyrrolidone solution. Along a network of PTFE rollers, the carbon filament was guided through the solution bath; at the outlet, extra solution was drained using a PTFE spinneret with a 12K filament outlet diameter of 1.0–1.1 mm, as can be seen in Figure 1.

The impregnated yarns were divided into 2 m long parts and dried for 24 h in a vertical position at a temperature of 23 ± 2 °C. The cured filaments were then cut into 230–330 mm long test specimens that were free of visible surface sagging of the polymer. The final solvent removal process took place in a Binder FD-115 oven (BINDER GmbH, Tuttlingen, Germany) for 6 to 8 h at 115°C.

The reference samples were carbon fibers impregnated with Sicomin SR1710 epoxy binder and SD 7820 hardener (Sicomin Epoxy Systems, Chateauneuf les Martigues, FRANCE). According to the scheme indicated in Figure 1, impregnation with a binder was carried out in a fluoroplastic tool. Due to the lengthy polymerization period and strength properties equivalent to Ultrason S 2010, the brand of epoxy binder used for vacuum infusion was chosen.

The yarns impregnated with epoxy resin were cut into fragments 2 m long, after which they were preliminarily cured at a temperature of 23 ± 2 °C, under a load of 1 kg, in a vertical position for 48 h. The filaments, cut into samples, were finally cured in a Binder FD-115 oven at a temperature of 115 °C for 6 h.

For tensile tests of the original carbon filaments, samples impregnated with thermoplastic and thermosetting binders, samples were used with ends sealed in protective cardboard frames.

Samples glued into pads made of cardboard 2 mm thick, 52 × 60 mm in size, with milled channels 0.4 mm deep and 1 mm wide were produced using Sicomin SR1710 epoxy binder with SD 7820 hardener.

**Figure 2:**
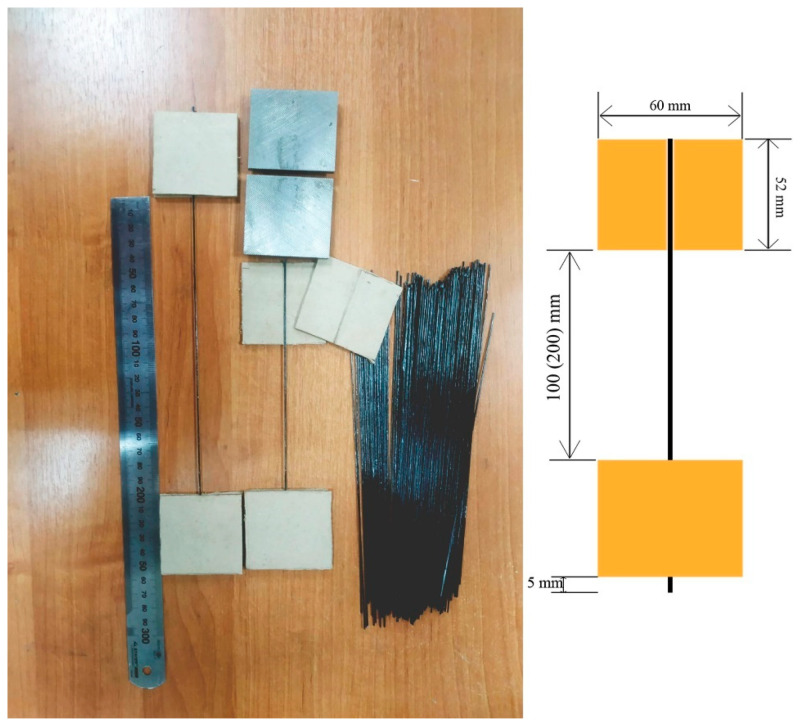
Samples and equipment for testing carbon filaments.

The dimensions of the cardboard overlays were chosen based on the geometry of the clamping jaws of the grippers of the universal testing machine used in the work. To polymerize the epoxy binder, the samples were placed in a Binder FD-115 oven and kept at a temperature of 115 °C for 6 h. Samples for testing and the equipment used are shown in Figure 2.

### 2.3. Characterization

Determination of the density of carbon fibers was carried out in accordance with ISO 1183-1:2019 Plastics—Methods for determining the density of non-cellular plastics—Part 2: Density gradient column method. 

The determination of the linear density of carbon filaments was carried out using ASTM D1907/D1907M–12 Standard Test Method for Linear Density of Yarn (Yarn Number) by the Skein Method, on samples 1 and 10 m long using an AND GR 202 analytical balance (AND, Japan).

The content of the polymer in microplastics was determined by the difference in the masses of yarns impregnated and not impregnated with polymer on samples 230–330 mm long before gluing them into cardboard frames using an AND GR 202 analytical balance (AND, Japan).

A Zwick/Roell Z020 universal tensile testing machine (Zwick/Roell Group, Ulm, Germany) with a maximum applied force of 20 kN, equipped with a high-precision MultiXtens contact strain measurement system, was used to determine the strength and deformation characteristics of carbon filaments impregnated with a thermoplastic polymer while considering the requirements of ASTM D4018 and ISO 10618.

Via a constant force of adhesion to the specimen, the MultiXtens system measures strain with polymer-tipped contact probes. At maximum active gripper travel speeds of up to 500 mm/min, the sensor enables strain measurement with an accuracy of 0.2 m. While maintaining a constant closing force and a pressure in the pneumatic cylinders equal to 3 bar, the samples were fixed using steel sponges with a size of 50 × 60 mm^2^ and a fine notch in pneumatic grippers of the vice type.

The working length of the yarns in the grips was 100 or 200 mm, the rate of active grip during the test varied from 1 to 100 mm/min, and the strain measurement base using the sensor was 70 or 150 mm, respectively. In the process of testing, the sliding of the sensor probes on the surface of the sample was not observed, and the destruction of the samples occurred in the working part. Taking into account the influence of the angle of orientation of the composite relative to the direction of application of the load, the deviations between the direction of orientation of the fiber and the direction of application of the load were not more than 1–2 ° on the test results when installing the samples.

Prior to the test, the samples were conditioned for 88 h at a standard atmosphere of 23/50 in accordance with ISO 291:2008 Plastics—Standard atmospheres for conditioning and testing. For each type of fiber, each test speed, and polymer concentration in the impregnating solution, 15 to 30 samples were tested. For each of the tested samples, the actual polymer content was calculated before it was framed.

The microstructure of the samples before and after testing was studied using scanning electron microscope TESCAN VEGA Compact (Joint stock company, TESCAN, Brno, Czech Republic).

## 3. Results and Discussion

**Figure 3:**
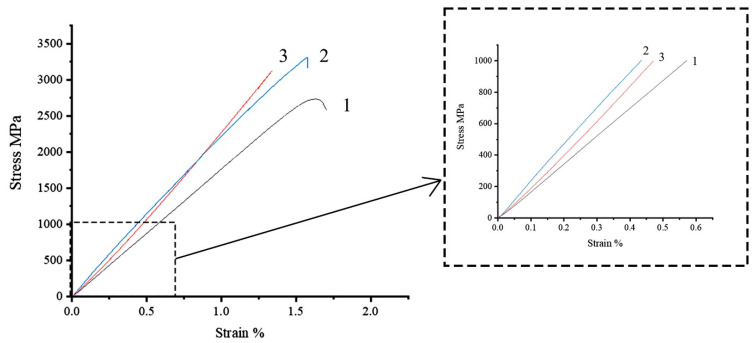
Strain–stress diagrams for samples based on high-strength fiber Toray T700. Test speed 10 mm/min. 1–unimpregnated fiber; 2–fiber impregnated with epoxy binder; 3–fiber impregnated with polysulfone.

Comparison of the tensile deformation behavior of high-strength Toray T700 12 K fibers impregnated with polysulfone and epoxy binder (70 wt.% of fibers and 30 wt% of polymer matrix), as well as unimpregnated filaments when tested with a constant speed of the active grip, is shown in Figure 3. Measurements of deformation were carried out using a contact sensor to exclude the influence of the testing system and the method of sample fixing on the results and the appearance of the diagram.

The diagram for an unimpregnated thread (Figure 3, curve 1) is linear until the beginning of the destruction of individual filaments, leading to the appearance of nonlinearity in the final section of the diagram. The nonlinearity of the initial section, which can appear due to the non-simultaneity entry into operation of individual bundles of filaments (that do not interact along the side surfaces), is expressed weakly. This indicates the good orientation of the individual filaments in the thread and a small number of destroyed filaments per length unit. The destruction occurred in the working part, while the difference in the strength of the thread determined without the use of a contact strain gauge and with the use of a contact gauge was not revealed, which allows us to conclude that the use of a contact gauge made of polymeric material when measuring strain does not damage the unimpregnated fiber. The destruction of the thread occurs within a short period of time and is accompanied by a significant increase in the volume of the sample.

The modulus of elasticity for microcomposites with an epoxy matrix is close to the passport values of 230 GPa, which indicates a high quality of impregnation and a high degree of implementation of fiber properties in such a composite. The initial section of the diagram is almost linear (Figure 3, curve 2); linearity is maintained up to stresses of up to 80% of the destructive one, when a slight nonlinearity appears due to the local destruction of individual fiber bundles and polymer microvolumes. The destruction occurs almost instantly with the separation of the sample into a large number of needle-like fragments. This type of load–strain curve and deformation behavior is typical for epoxy reinforced with Toray T700 fiber [51,52].

The deformation of fiber impregnated with a thermoplastic matrix differs from the behavior of similar samples with an epoxy one (Figure 3, curve 3). The diagram contains a significant initial nonlinear section in the stress range up to 200–220 MPa, within which the elastic modulus increases from 120 to 230 GPa. The second stage of the increase in the values of the elasticity modulus lies at stresses above 1200–1500 MPa. Such behavior allows us to suggest that individual bundles of filaments in the samples are not uniformly loaded at the initial stage. After the yield point of polysulfone is reached, the polysulfone begins to flow in the direction of applied load application, which provides the possibility of limited mutual sliding and alignment of individual bundles of filaments in the fiber, which allows for achieving a high degree of their orientation at stress values close to the tensile strength. This ensures the achievement of high values of the modulus of elasticity of the fiber impregnated with thermoplastics.

The destruction of samples with a thermoplastic matrix occurred in the working part, almost instantly, while, unlike samples with an epoxy matrix, the sample did not crumble, but was divided into separate fragments that retained a connection with each other.

For samples based on high-strength fibers UMT49 and high-modulus UMT400, a similar type of load–strain diagrams and patterns of their change are observed. Differences in the form of load–strain diagrams and breaking load for samples with different lengths of the working part (100 and 200 mm) and different strain measurement bases were not revealed; therefore, we obtained the main data array on samples 100 mm long with a strain measurement base of 70 mm.

The deformation behavior of composites with a thermoplastic matrix is affected by the rate of application of the load and the content of the polymer forming the matrix. We studied this effect, for which the content of the polymer binder was determined in each sample of the composite according to the known values of the linear density of the used threads and mass for each single sample. We changed the load rate by adjusting the active grip movement speed, which we changed in the range from 1 to 100 mm/min.

**Figure 4:**
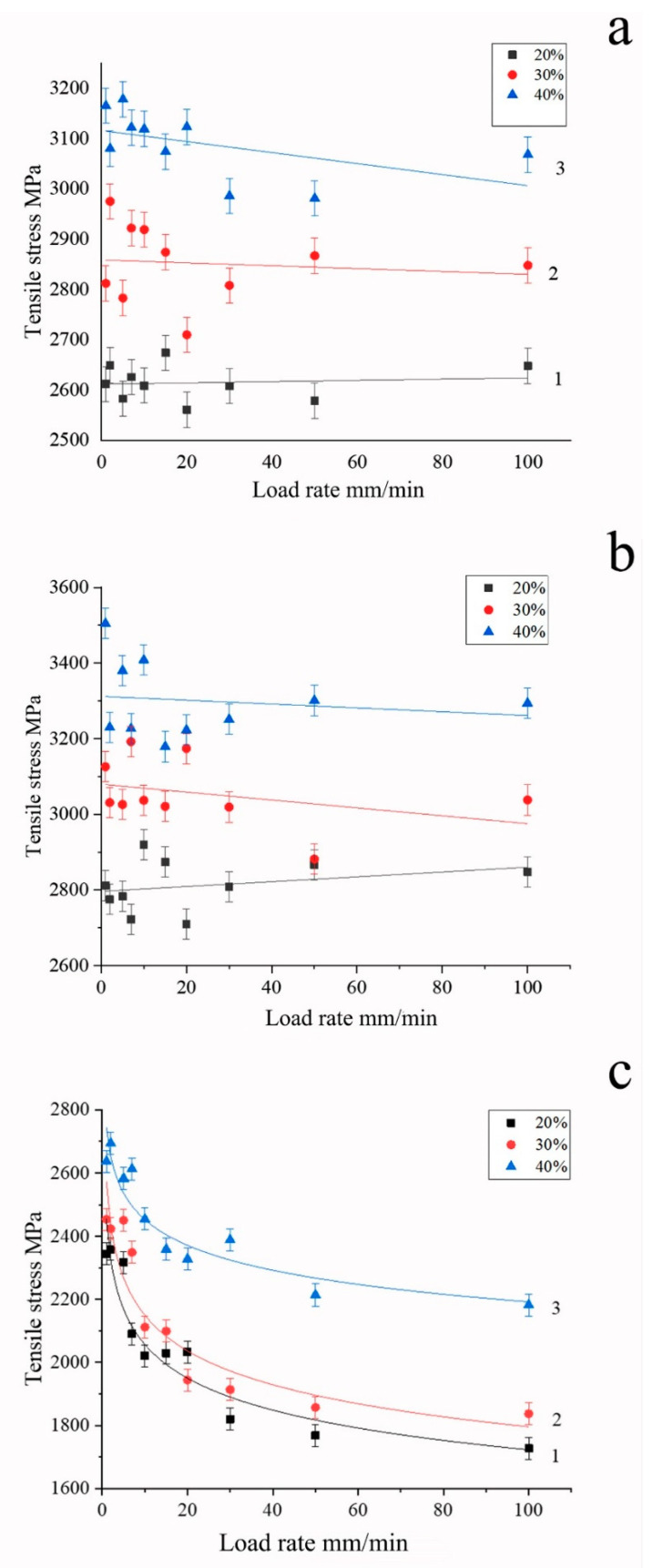
Dependences of the tensile strength on the loading rate for composites based on Toray T700 (**a**), UMT49 (**b**), and UMT400 (**c**) carbon fibers, polysulfone content indicated in wt. %.

**Figure 5:**
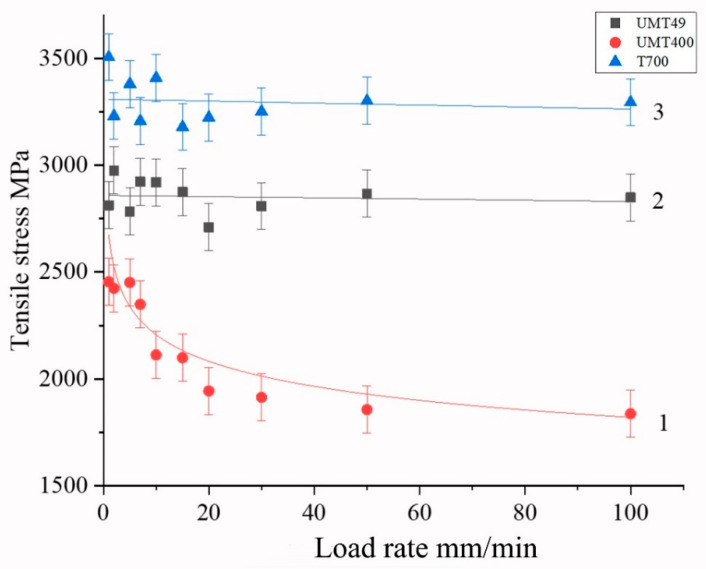
Dependences of tensile strength on the loading rate for composites containing 30 wt. % of polysulfone.

**Figure 6:**
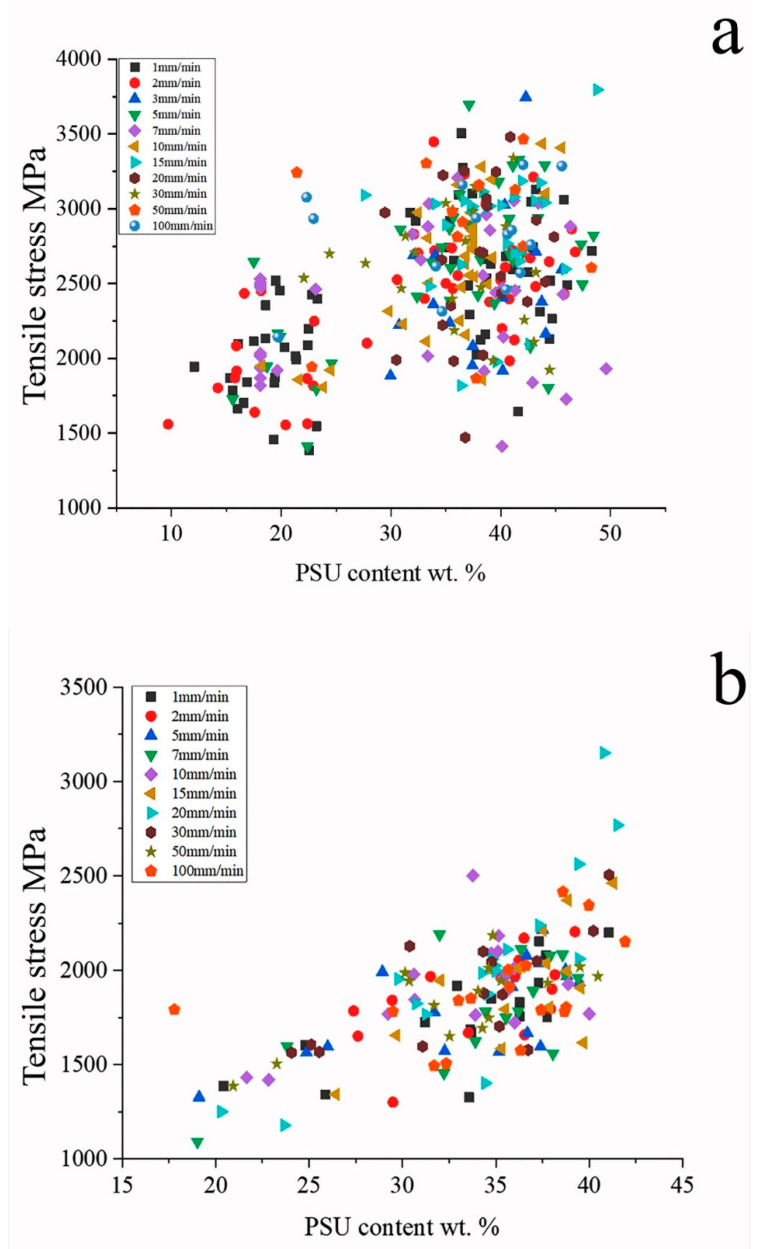
Dependences of tensile strength on the polysulfone content for Toray T700 (**a**) and UMT400 (**b**) based composites at various loading rates (indicated in figure).

**Figure 7:**
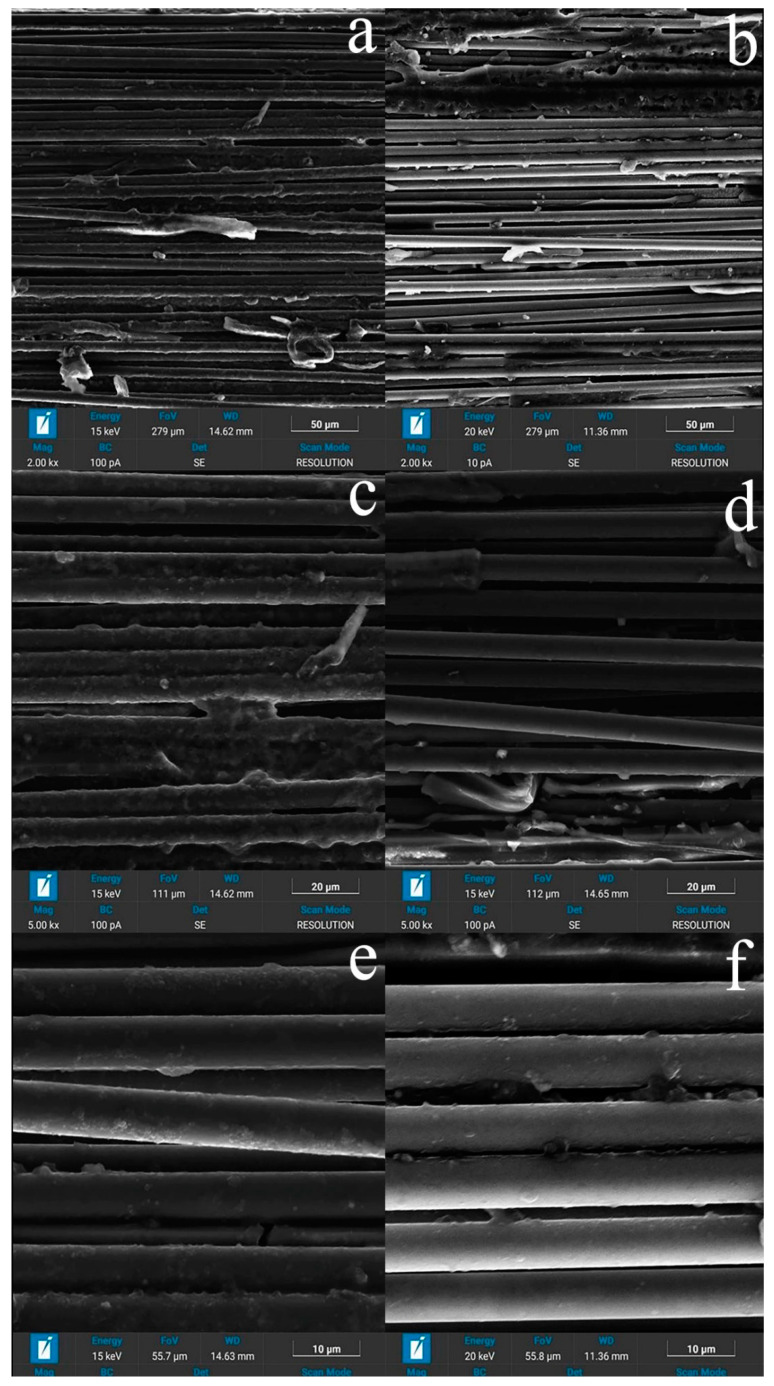
Surface destruction of the microcomposite CF–PSU. (**a**,**c**,**e**) Toray T700 high-strength fiber (**b**,**d**,**f**) UMT400 high-modulus fiber.

The obtained dependences between the speed of movement of the active grip and the polymer to fiber ratio and the tensile strength are shown in Figure 4, Figure 5, Figure 6 and Figure 7 and in Table 2.

To plot dependencies given in Figure 4 and Figure 5, samples with content of polysulfone not differing by more than ± 2 wt.% from the base values of 20, 30, and 40 wt.% were used. For high-strength Toray T700 and UMT 49 fibers, the test speed had little effect on the magnitude of the breaking stresses in the investigated speed range (Figure 4a,b). Similar behavior of Toray T700 fibers was noted by authors of [52], who studied the effect of test speed for epoxy matrix single-fiber composites and observed that test speed had little effect on the tensile strength and elasticity modulus. Based on the fiber bundle model and the statistical theory of fiber strength, they developed a constitutive damage model based on the Weibull distribution function.

Polymer content in samples has a much greater effect on the tensile strength, which is associated with the distribution of the polymer inside the sample and the nature of its interaction with the fibers, as will be considered below. With an increase in the polymer content, we observe an increase in the tensile strength in the entire range of the considered concentrations, which is practically independent of the loading rate, both for high-strength and high-modulus fibers (Figure 4). It should be noted the highest value of tensile strength was achieved at a polymer content of about 40 wt.%.

For more brittle, high-modulus graphitized fibers UMT400, there is a noticeable effect of the load application rate on the fracture strength (Figure 4c). With an increase in the load, the strength of the microcomposite drops from 2400–2700 to 1900–2400 MPa at a load rate of 30 mm/min. This is due to damage of brittle filaments during the polymer flow. The highest value of tensile strength of composites based on high-modulus fibers was also achieved at the concentration of polysulfone of 40 wt.%; however, for such composites, the tensile strength increases with an increase in fiber content much higher than for the high-strength one. It should be noted that the tensile strength values for high-strength carbon fiber, given in Figure 4a,b, are significantly higher than those obtained in [49] for high-strength carbon fiber impregnated with polysulfone using melt technology. This means that the solution impregnation method allows for achieving a significantly higher quality of composites than the melt impregnation technique. 

Figure 5 illustrates the difference between mechanical behavior of various carbon fiber types in polysulfone-based composites, showing that, whereas for high-strength fibers the dependence of tensile strength is nearly not revealed, for high-modulus fibers, the tensile strength values exhibit a pronounced logarithmic dependence on the loading rate. 

A decrease in strength with an increase in the fiber content in a thermoplastic composite based on polypropylene reinforced with glass and carbon fibers was noted in [53]. This decrease was attributed to a decrease in the average fiber length. It was noted that, in terms of the impact on strength, the fiber efficiency ratios decreased with increasing fiber volume fraction, and the more brittle fiber, namely, carbon fiber, corresponded to a lower ratio than glass fiber [53].

Figure 6 presents the dispersion of the results illustrating the correlation between tensile strength of composites containing high-strength and high-modulus fibers and the polymer content in samples. The method of sample preparation by the solution technology leads to an almost continuous distribution of the thermoplastic content in the composite, the data shown in Figure 6 allow us to obtain a refined form of the dependences. It is seen that concentration dependence is revealed as stronger for high-modulus fiber-based composites than for high-strength fiber-based ones.

**Figure 8:**
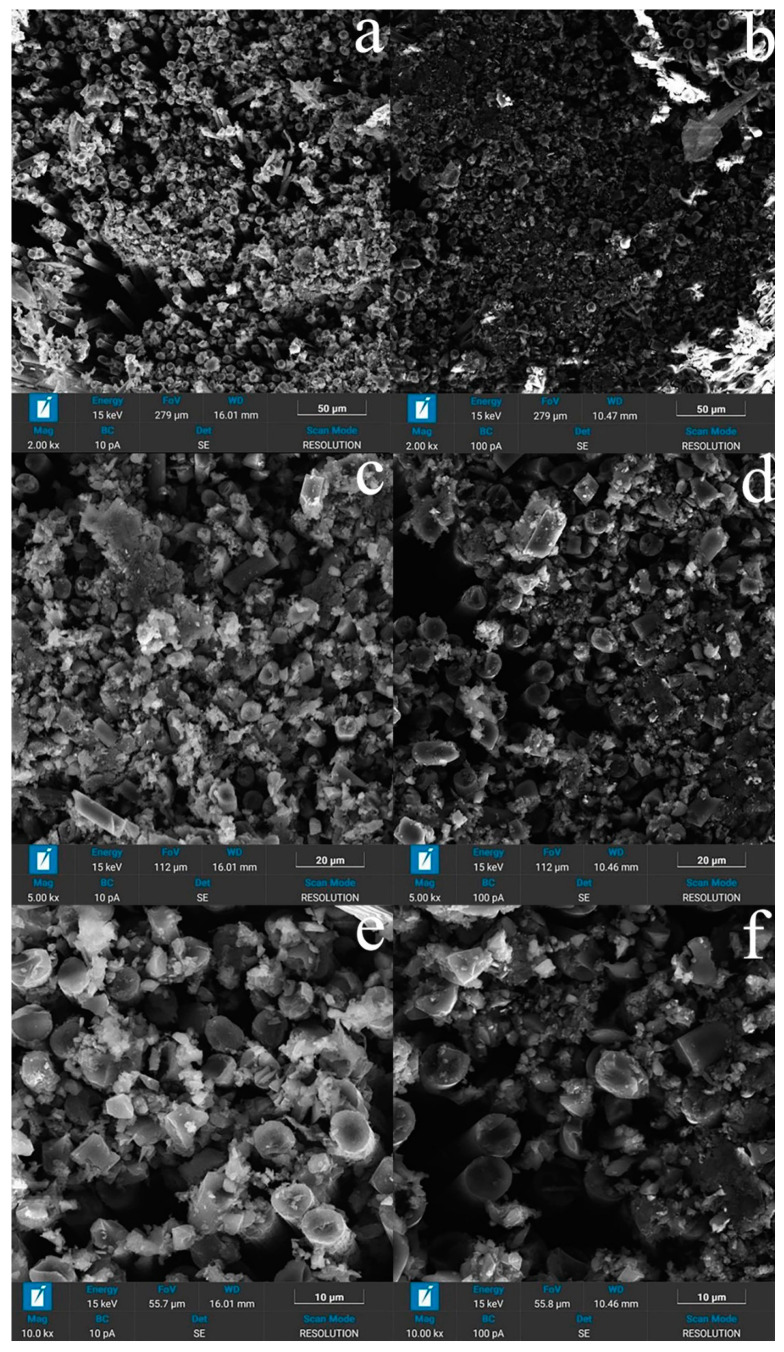
Surface destruction of the microcomposite CF–PSU. (**a**,**c**,**e**) Toray T700 high-strength fiber (**b**,**d**,**f**) UMT400 high-modulus fiber.

The observed patterns of composites’ deformation can be explained using the data of scanning electron microscopy presented in Figure 7 and Figure 8. The difference in the processes of used carbon fibers’ fabrication, which consists of the maximum temperatures for their processing—as a rule, values less than 1700 °C for high-strength fibers and above 2000 °C for high-modulus fibers—leads to different chemical activity of their surfaces. The level of adhesive interaction between polysulfone and high-modulus fibers (Figure 7b,d,f) is lower than between polysulfone and high-strength fibers (Figure 7a,c,e), and the distribution of the polymer over the cross-section of the rods is non-uniform (Figure 8). The surface layer of the fibers contains a larger amount of polymer, and the polymer itself covers the surface of individual filaments with a thin layer and forms interlayers up to 5-7 μm thick between individual bundles of filaments.

During the destruction of samples with a thermoplastic matrix, they were divided into separate fragments that retained a connection with each other. The number of damaged filaments in the case of high-modulus samples is greater than for high-strength ones (Figure 7a,b), which confirms the assumption that more brittle high-modulus fibers are more susceptible to damage. During the destruction of the samples, a thin polymer film of 0.2–0.3 μm in thickness remained on the surface of high-strength fibers, which, in our opinion, represents the remaining connected transition layer that existed at the polymer–matrix interface in the original composite. On individual fragments of high-modulus fibers, thin interlayers of the polymer bound to the fiber surface were observed, but due to the lower chemical activity of the surface of high-modulus fibers, the frequency of their appearance was much lower than for high-strength fibers, and a significant part of the fiber surface at the time of destruction was not connected with the polymer.

Based on the analysis of the structure presented on the SEM, the process of matrix formation consists of successive processes, the course of which depends on the amount of polymer in the composite and differs in the middle part of the thread and on the surface.

Inside the thread, at the first stage of impregnation, a thin film of polysulfone is formed on the surface of single filaments, the thickness of which reaches 2 μm (Figure 7d). Further, as the solvent is removed, thin films are formed that connect individual adjacent filaments into bundles and form polysulfone interlayers up to 5-7 microns thick. At the third stage, the extended pores located between individual filaments and bundles are filled (Figure 7c).

In addition, in samples with a polymer concentration of more than 30 wt. % on the surface of a composite sample, a near-surface zone 100 μm thick is usually observed, enriched with polymer covering the entire rod as a whole, while extended pores can remain in the middle part. In samples with a low polymer content (less than 30 wt %), polysulfone becomes insufficient to completely fill the extended pores between the bundles (Figure 7a,b). In addition, in the structure of polymer layers with a thickness of more than 2 μm, one can distinguish spherical pores formed during the evaporation of the solvent.

Thus, based on the SEM data, the decrease in strength with a decrease in the content of polysulfone in the composite, in our opinion, is primarily associated with a decrease in the amount of polymer, which becomes insufficient to completely fill the extended pores between the bundles and individual filaments. This leads to a deterioration in the redistribution of mechanical stresses between the individual parts of the composite.

Thus, at the tension of the samples, we have a certain amount of material volume in which the fibers are not sufficiently bonded along the side surfaces and have the ability to slide relative to each other. With the increase in the applied load, the processes of alignment and orientation of filaments and bundles of filaments in the direction of the load proceed, and the elastic modulus increases to values of 220–230 MPa. Numerous areas of the polymer matrix elongated and oriented in the direction of the applied load are observed on the fracture surfaces (Figure 7a–d).

The cross-section of the destroyed samples based on high-strength and high-modulus fibers is shown in Figure 8. In the process of destruction, fragmentation of the material occurs with the preservation of dense, slightly damaged bundles of filaments separated by cracks in the polymer matrix (Figure 7a,b). It is also clearly seen that the adhesive interaction at the high-modulus fiber–polymer interface is lower, and a lower amount of bounded polymer appears on it than for high-strength ones (Figure 7c–f.).

The appearance of intra-strand damage and their complex nature using the X-ray computed tomography method are considered, for example, in [54], and the tendency of thermoplastic polymers to orientational stretching inside the fibers impregnated with poly ether ether ketone, similarly to those observed in our study, was shown by the authors of the work [46].

Comparison of composite materials with epoxy matrices of the polysulfone-based composite studied by us showed a difference in the nature of destruction in the zone of the fiber–matrix interface. In the case of epoxy binders [33], the fracture occurs along the interface, in contrast to our composite, in which the fracture occurs along the polymer and the transition layer material remains in contact with the fiber surface.

It is also important to note that in composites based on thermosets, matrix damage that occurs before the onset of intense destruction is local, not accompanied by intense destruction of fibers [38,39]. Similar to [44], in which PEEK-CF composites and CF epoxy resin are considered, polysulfone–CF composite materials show an advantage due to the microscale shear deformation implemented in them. This is also due to the high interfacial strength of the fiber matrix until the final destruction of the elastomer, in contrast to extensive microcracks, and disruption of the fiber–matrix bond in the epoxy composite.

## 4. Conclusions

Our studies have shown that the deformation behavior of carbon fibers impregnated with thermoplastic polymers, such as polysulfone, differs from that characteristic of composites with thermosetting matrices. There are two nonlinear regions in the load–strain diagrams. The nonlinearity of the initial section is associated with the sliding of the bundles of filaments of the fiber relative to each other, leading to the orientation of the filaments in the direction of the load application, accompanied by an increase in the elastic modulus from 120 to 230 GPa. The second nonlinear section occurs at stresses above 1200-1500 MPa and is caused by the destruction of the extremely elongated polymer interlayers, which leads to a further increase in the elastic modulus to 250 and more GPa at the samples’ destruction.

The test speed has practically no effect on the tensile strength of unidirectional composites with high-strength carbon fibers, while, for high-modulus fibers, a decrease in strength by 30% is observed when the loading speed changes from 1 to 30 mm/min.

With an increase in the polymer content from 20 to 40 wt.% of polysulfone, the strength of composites increases by 35% for high-strength fibers and up to 50% for high-modulus carbon fibers. Such an increase is due to the structure of the polymer distribution in composites, in which, with an increase in polymer content, pores disappear in the interfilament space, which arise due to the characteristics of impregnation with polymer solutions.

## Figures and Tables

**Table 1:** Characteristics of carbon fibers used in this study (manufacturer’s technical data).

Fiber Grade	Number of Filaments	Filament Diameter, µm	Strength, GPa	Elastic Modulus, GPa	Deformation Limit, %
T700SC	12,000	7.0	4.9	230	2.1
UMT49	12,000	7.0	4.9	260	1.8
UMT400	12,000	6.5	4.2	400	1.1

**Table 2:** Dependence of the strength of carbon fiber–polysulfone composites on the loading rate and polysulfone content.

Loading Rate, mm/min	T700			UMT49			UMT400		
	PSU Content, wt. %			PSU Content, wt. %			PSU Content, wt. %		
	20%	30%	40%	20%	30%	40%	20%	30%	40%
1	2812 ± 116	3505 ± 124	3126 ± 111	2612 ± 107	2812 ± 119	3165 ± 94	2345 ± 96	2454 ± 125	2637 ± 141
2	2775 ± 109	3230 ± 140	3031 ± 113	2649 ± 113	2975 ± 104	3080 ± 136	2359 ± 113	2424 ± 101	2694 ± 103
5	2783 ± 129	3380 ± 143	3026 ± 95	2583 ± 106	2783 ± 108	3178 ± 88	2317 ± 95	2451 ± 104	2583 ± 134
7	2722 ± 130	3227 ± 103	3192 ± 92	2626 ± 130	2922 ± 120	3122 ± 137	2090 ± 91	2349 ± 121	2613 ± 94
10	2919 ± 106	3408 ± 136	3037 ± 115	2609 ± 91	2919 ± 108	3119 ± 113	2021 ± 87	2112 ± 135	2455 ± 125
15	2874 ± 108	3179 ± 122	3021 ± 126	2674 ± 125	2874 ± 102	3074 ± 125	2029 ± 125	2099 ± 123	2359 ± 97
20	2710 ± 163	3223 ± 89	3174 ± 88	2561 ± 123	2710 ± 95	3123 ± 96	2033 ± 112	1944 ± 81	2328 ± 94
30	2808 ± 112	3251 ± 115	3019 ± 114	2608 ± 99	2808 ± 123	2986 ± 106	1820 ± 107	1914 ± 114	2389 ± 76
50	2867 ± 122	3301 ± 95	2882 ± 112	2579 ± 97	2867 ± 104	2981 ± 78	1768 ± 105	1857 ± 91	2214 ± 161
100	2848 ± 118	3294 ± 103	3038 ± 109	2648 ± 131	2848 ± 78	3068 ± 117	1727 ± 89	1837 ± 153	2182 ± 81

## Data Availability

The data presented in this study are available on request from the corresponding author.
